# Health Diaries to Empower Marginalized Communities Through Self-Tracking: A Study in an Urban Slum and Rural Area of Bhopal District, Madhya Pradesh

**DOI:** 10.7759/cureus.92900

**Published:** 2025-09-22

**Authors:** Devendra Gour, Manju Toppo, Madhav Bansal, Indu Jyotsana Ekka, Durgesh Sharma, Neeta Kumar, Pallavi Mandrai, Gursharan Singh Mehta

**Affiliations:** 1 Community Medicine, Gandhi Medical College, Bhopal, IND; 2 Community Medicine, Gandhi Medical College, Bhopal , IND; 3 Public Health Cancer Nutrition, Indian Council of Medical Research, New Delhi, IND

**Keywords:** healthcare utilization, health diaries, health literacy, health surveillance, home health guides

## Abstract

Background and objective: Health equity requires reliable, community-level data to inform policy and service delivery. In resource-constrained settings such as urban slums and rural areas of India, formal health data systems are often inadequate. This study aimed to assess the acceptability and use of self-maintained health diaries for community-based health surveillance.

Methods: A cross-sectional study was conducted in two demographically distinct underserved blocks in Bhopal district, Madhya Pradesh, India, covering 1132 urban slum and 1162 rural residents. Households were provided monthly health diaries to self-record illness episodes, healthcare utilization, and basic health parameters with support from home health guides (HHGs). Baseline and follow-up data were collected over multiple months using structured surveys and biometric screenings. Baseline data were collected initially by the respective HHG, study staff, and local health volunteers. The baseline data included sociodemographic characteristics, anthropometric measurements, blood pressure, screening for common illnesses and disorders, health needs, availability and cost of care, affordability of health care services, population distribution, health services distribution, health-seeking behavior of the community via their preferences and reasons, diary acceptability, availability and utilization of existing health care facilities, and knowledge of the community. Data was entered and analyzed in Microsoft Excel (Microsoft Corp., Redmond, WA, USA).

Results: Diary compliance was high across both settings. In the urban slum, illness reporting rose from 122 (10.4%) to 142 (12.5%), while in the rural area, it declined from 89 (7.6%) to 19 (1.6%). Significant reductions in health expenditures were observed post-intervention, with urban lab test costs dropping from 418 (36.9%) to four (0.3%) and rural costs from 235 (20.2%) to two (0.2%). The use of the health diary also enhanced early detection of anemia, pre-hypertension, and pre-diabetes. Diaries enabled the reporting of diverse conditions, from headaches and menstrual disorders to otitis media and stroke, highlighting their value in capturing a wide disease burden.

Conclusion: Self-maintained health diaries are feasible, acceptable, and cost-effective tools for improving health literacy, surveillance, and health-seeking behavior in underserved populations. Integration into national health programs and digital platforms could amplify their impact.

## Introduction

Making health equity a reality for all and moving towards the progressive realization of human rights requires that all individuals have access to quality health care. Health-seeking behavior is a multi-dimensional concept and depends on the context and the individual. Many factors are associated with health-seeking behavior, namely the type of sickness, degree of illness, gender, surrounding social environment, cost of care, social beliefs about the cause of illness, quality of care, education, and economic background. The healthcare infrastructure of a country determines the health-seeking behavior of that country’s population [1].

The proper goal for any healthcare delivery system is to improve the value delivered to patients. It is not the number of different services provided or the volume of services delivered that matters, but the value. To properly manage value, both outcomes and costs must be measured at the patient level. Measured outcomes and costs must encompass the entire cycle of care for the patient’s particular medical condition, which often involves a team with multiple specialties performing multiple interventions from diagnosis to treatment to ongoing management [2].

Achieving health equity requires widely encompassing and up-to-date data on the health conditions of a population; without data, policymakers cannot determine the effect of interventions on public health and inequalities. However, inadequate funding and lower priority, particularly in low- and middle-income countries, mean that such data are not always available [3-5].

To bridge this data gap, community participation in the generation of health data has been identified as a core value of authentic health impact assessment [3]. Health diaries are personalized records and a proactive method for individuals to monitor their health status and health-related information, track symptoms, and adhere to medication regimens. The benefits of health diaries are promoting self-management, facilitating communication with healthcare providers, and improving health outcomes among people. By harnessing the power of health diaries, healthcare systems can empower people to take an active role in managing their health, foster collaborative partnerships between patients and providers, and ultimately contribute to better healthcare engagement and outcomes in populations. The disparity in healthcare access between rural and urban areas necessitates urgent action to enhance healthcare infrastructure, train healthcare personnel effectively, and implement robust public health initiatives [6-9].

India's commitment to achieving universal health coverage by 2030 is commendable and pivotal in addressing these discrepancies. To realize this goal, substantial investments in healthcare infrastructure, comprehensive training programs for healthcare professionals, and initiatives to promote preventive healthcare measures are imperative. Digital health interventions with support or through training of grassroots-level health workers can help in bridging the gap in the delivery of primary health care [10]. But factors like infrastructure, affordability, family income, education, and occupation restrict the availability and use of digital tools [11]. In these circumstances, health diaries build upon principles of patient-centered care and self-management, emphasizing the importance of tailoring healthcare interventions to individual needs and preferences.

Handbooks for maintaining maternal and child health home-based records (HBRs) were found to be used by health providers and mothers across all quintiles of the poorer population in Afghanistan [11]. The HBRs facilitate caregiver education, coordination of care, and public health monitoring and reporting [12,13]. Through regular use of health diaries, adults can gain insights into their health patterns, identify triggers for symptoms or exacerbations, and make informed decisions about their care and their families [14]. Health diaries facilitate communication with healthcare providers by providing a comprehensive overview of the patient's health status and treatment history, enabling more personalized and effective care delivery [15].

To generate more complete health data for impoverished communities (urban slums and rural) in India, we investigated the possibility of community members maintaining a regular health diary. We aimed to determine the acceptability of such an intervention among members of these communities, as well as its feasibility in terms of helping with the periodic updating of the diary, where necessary, and retrieving recorded data. Through this study, we assess the acceptability and use of self-maintained health diaries for community-based health surveillance.

This study is part of the Indian Council of Medical Research (ICMR)-funded study titled 'Task Force Study for Evaluation of Community Level Acceptability, Scalability, and Linkage Within the Health System of ICMR Pre-validated Labike/Technologies for Screening and Diagnosis in Rural and Urban Population: An Implementation Research'.

## Materials and methods

**Figure 1:**
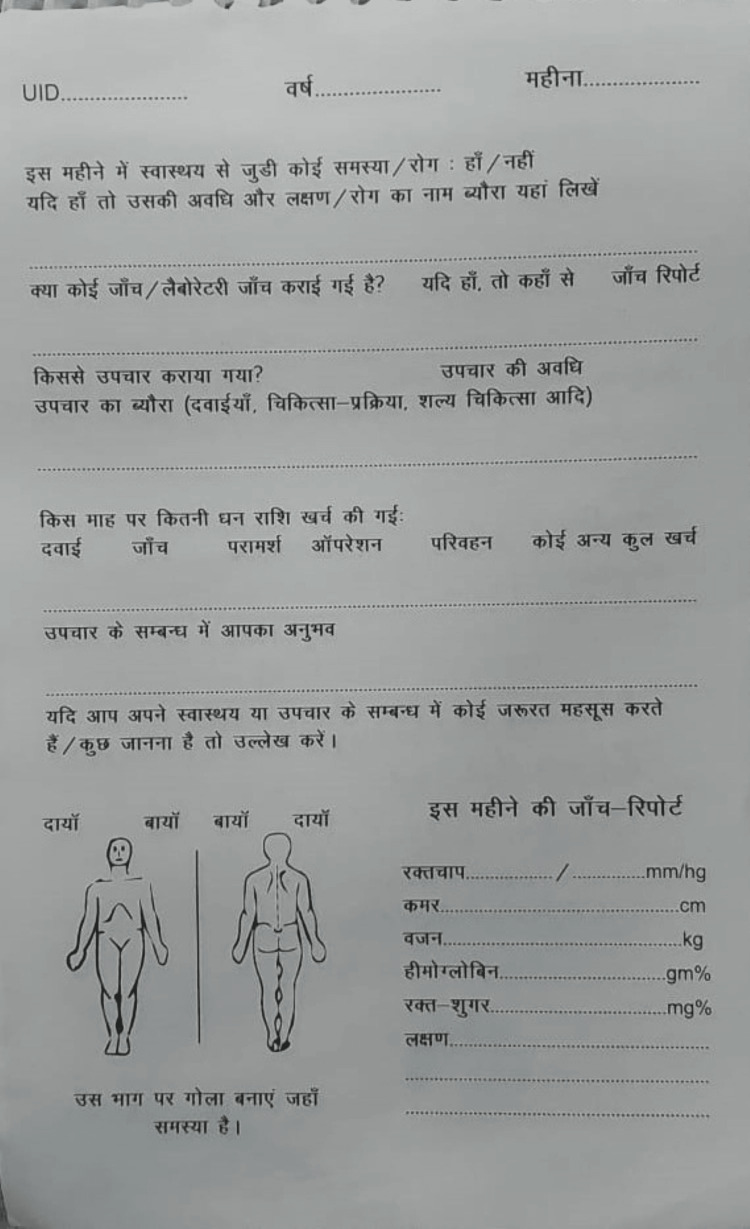
Sample page of the health diary in Hindi UID: Unique identification

**Figure 2:**
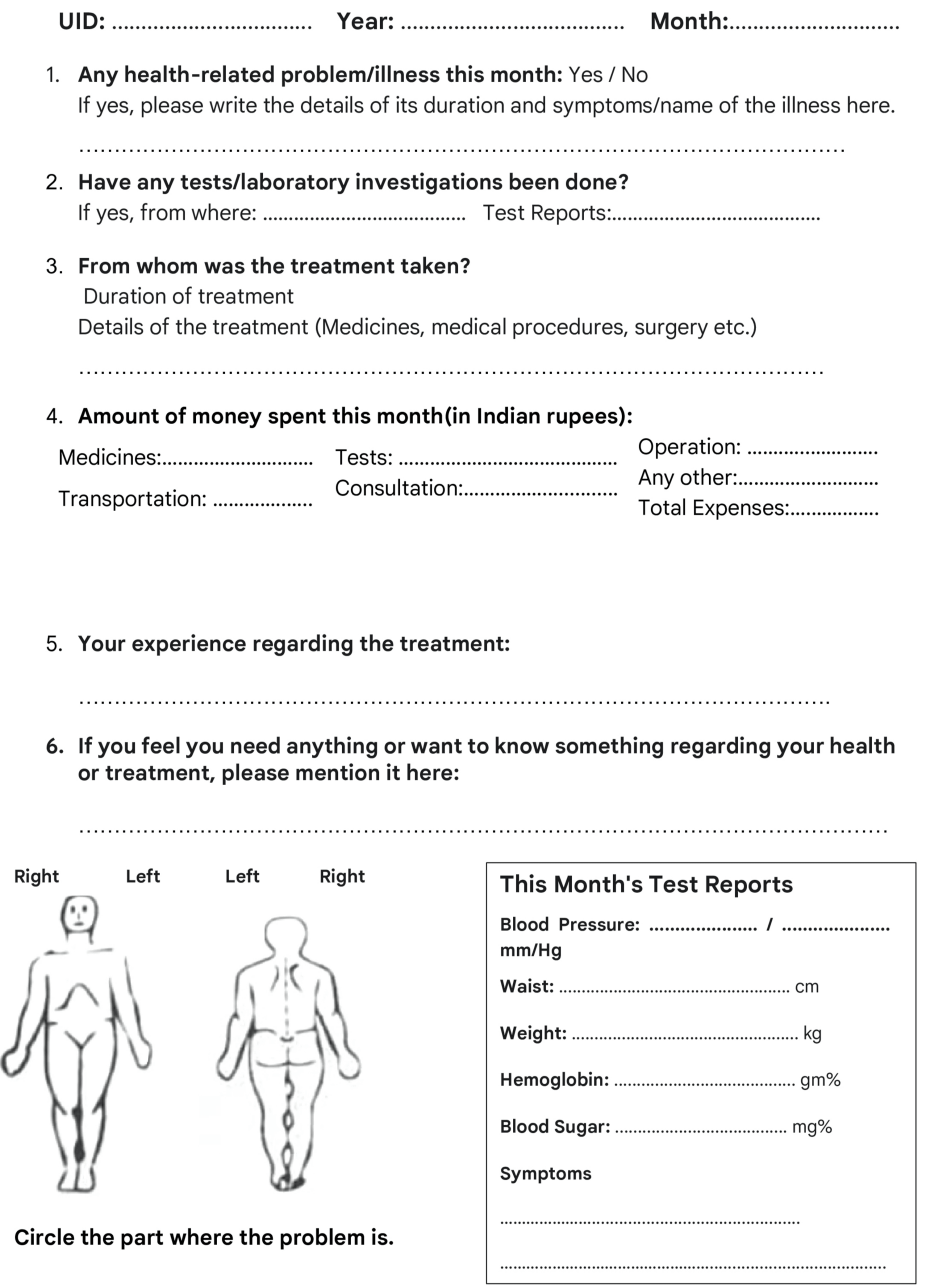
Translation of the sample page UID: Unique identification

This study was conducted in two underserved blocks of a Bhopal district in Madhya Pradesh, India. The Institutional Ethics Committee of Gandhi Medical College (Bhopal, MP, IND) approved the study (approval no. 6699/MC/IEC/2022). Two impoverished areas, i.e., areas with poor socioeconomic conditions, one from a rural area and the other from an urban slum, were identified for this study. After a wide discussion with public health experts and social scientists, the ICMR designed a health diary for communities. Each page features pertinent questions in Hindi that are to be filled in for each month and is accompanied by a carbon duplicate (Figure 1). The translation of the sample page is featured in Figure 2.

Space to record the participant’s unique identification (UID) number was provided on each page. We selected an update frequency of one month based on an acceptable period for recall. Although shorter update periods would reduce recall bias, they may also have resulted in reduced compliance.

Each team was provided with a laptop, screening equipment for measurement of height, weight, blood pressure, glucose, and hemoglobin, and health diaries for distribution. The households within each site were randomly chosen. Each study team ensured its sample was representative by checking that the households spanned both central and peripheral areas of its study site. A baseline survey for demographic profiling was conducted by each team on a door-to-door basis. All members of the teams involved in the survey were trained with the assistance of a coordinating team.

In addition to basic demographic information on education level, employment, and income, information on the frequency of healthcare seeking of the community members and healthcare expenses incurred was collected. All baseline interviews were conducted with the designated main guardian of each family, who agreed to keep a health diary and provided consent for all members of the household younger than 18 years.

Health diaries were distributed to participants, and they were guided in completing each month’s page during brief and informal doorstep exchanges. Accredited Social Health Activists were oriented, briefed, and sensitized to help the family head/member fill the diary page on basic healthcare if they seek assistance for the same, and to collect the duplicate carbon pages. Health volunteers or the home health guide (HHG) recorded symptoms as reported by the diary holders and validated any actual diagnoses with available medical documents (e.g., a note or receipt from the doctor, caregiver, or pharmacist, or packaging from the prescribed medicine). The study participants were asked about the factors affecting the maintenance of home-based health records in the given diary, and their feedback was sought on the same. 

Study design

This is a cross-sectional door-to-door survey to record health data and cohort components via the intervention block that is followed up for monthly health updates. All the family members of participating families were included in the study, for which the head of the family was contacted to obtain consent. According to the 2011 census, two sites, one each from rural and urban underprivileged populations, were selected. Study blocks were selected from the enlisted blocks by the probability proportional to size (PPS) method. 

Data collection

Staff appointment was done for data collection. Local candidates for lab technicians and residents who were ready to become certified, self-employed HHGs for 50 houses were considered for selection. A total of 15 HHGs and 8 lab technicians were selected. They were oriented to the objective and methodology of the project and trained for data collection. Purchase and placement of the 'lab on bike' (LABIKE) with all features for online health data management was done. Health diary distribution was done by the study team with the help of HHGs to households having individuals with health IDs in the intervention block. Baseline data was collected digitally. The data also included anthropometric measurements and blood pressure. Blood samples were collected in the field to assess baseline biochemical parameters. The samples were tested using two LABIKES per site. The HHGs ensured that health diaries were updated monthly for illnesses. One HHG was responsible for 50 households.

Baseline data were collected initially by the respective HHG, study staff, and local health volunteers. The baseline data included sociodemographic characteristics, anthropometric measurements, blood pressure, screening for common illnesses and disorders, health needs, availability and cost of care, affordability of health care services, population distribution, health services distribution, health-seeking behavior of the community via their preferences and reasons, diary acceptability, availability and utilization of existing health care facilities, and knowledge of the community. This data was entered online. The HHGs collected the copy (carbon page) from the diary every month. Data from the monthly diary page featuring the morbidity data, including the cost of investigations and treatment, if any, and the reports of tests conducted by the LABIKE, were entered online. 

## Results

A total of 5000 health diary users participated in our study. The proportion of community members from the urban slum who agreed to participate in the diary project was 1327 (53.0%), and 1353 (54.1%) from the rural site. Of those who agreed to take part in our study, we observed that the proportion of partly completed diaries retrieved at the end of the year was 1132 (85.3%) at the urban site and 1162 (85.8%) at the rural site. We demonstrate how compliance with our project varied over the year-long period of diary page collection at both sites. Compliance was highest at 1250 (100.0%) and lowest with 1132 (90.5%) at the urban site and 1162 (92.9%) in the rural area. We recorded user satisfaction and willingness to continue maintaining the health diary among 44.7% of the participants. Participants assessed our study to be the best for increasing their awareness of other schemes; study staff and HHGs were not only providing education on preventive health care but, during their regular visits, were also keeping participants informed of other relevant interventions. There is a general trend of decreasing diary retrieval numbers over time in both urban and rural settings. In the initial months, the number of diaries retrieved was similar in both settings. However, the decline in diary retrieval appears to be more pronounced in the urban setting, leading to a lower number of diaries retrieved at the end of the study compared to the rural setting (Table 1).

**Table 1:** Number of health diary pages collected each month from both sites

Months	No. of pages collected from the urban site	No. of pages collected from the rural site
1	1250	1250
2	1247	1249
3	1249	1230
4	1229	1224
5	1207	1202
6	1195	1204
7	1165	1180
8	1152	1177
9	1155	1165
10	1137	1168
11	1135	1167
12	1132	1162

The findings from this study provide valuable insights into the demographic, clinical, and economic profiles of the population surveyed in the urban slum and rural settings of Bhopal. The demographic distribution (Table 2) showed a nearly equal gender representation, with 54.2% females in the rural area and 51.3% (581) in the urban slum. A large proportion of participants were either unemployed, homemakers, or students: 736 (63.3%) in the rural site and 729 (64.3%) in the urban site. Literacy levels were low, with 413 (35.5%) illiteracy in the rural area compared to 399 (35.2%) in the urban slum, highlighting an educational divide. A higher proportion of unskilled workers was observed in the rural population: 203 (17.4%) compared to the 150 (13.2%) from the urban population. Economically, 1154 (99.3%) of rural and 1110 (98%) of urban households earned less than ₹6,293/month, underscoring economic vulnerability in both settings. 

**Table 2:** Demographic profile of participants in the study

Demographic parameter		Urban n (%)	Rural n (%)
Sex	Male	551 (48.6)	532 (45.7)
	Female	581 (51.3)	630 (54.2)
Age (year)	0-9	251 (22.1)	202 (17.3)
	10-17	240 (21.2)	243 (20.9)
	18-24	204 (18.0)	240 (20.6)
	25-39	244 (21.5)	258 (22.2)
	40-64	136 (12.0)	142 (12.2)
	>65	24 (2.1)	32 (2.7)
	Unknown	1 (0.08)	3 (0.2)
Education	Illiterate	399 (35.2)	413 (35.5)
	Primary	332 (29.3)	368 (31.6)
	Middle school	171 (15.1)	163 (14.0)
	High school	96 (8.4)	106 (9.1)
	Intermediate	40 (3.5)	53 (4.5)
	Graduate	29 (2.5)	34 (2.9)
	Professional/honours/postgraduate	2 (0.1)	2 (0.1)
	Unknown	1 (0.08)	1 (0.08)
Occupation	Professional	3 (0.2)	1 (0.08)
	Semi-skilled	117 (10.3)	132 (11.3)
	Skilled	40 (3.5)	57 (4.9)
	Unemployed/homemaker/student	729 (64.3)	736 (63.3)
	Unskilled worker	150 (13.2)	203 (17.4)
	Unknown	3 (0.2)	1 (0.08)
Monthly family income	< 6293	1110 (98.0)	1154 (99.3)
	6294–18,858	21 (1.8)	8 (0.6)

Illness reporting and health expenditure trends revealed an increase in the urban slum from 122 (10.7%) to 142 (12.5%) post-health diary introduction, suggesting improved awareness and tracking. In contrast, rural illness reporting dropped from 89 (7.6%) to 19 (1.6%), which may indicate the most commonly reported reason for not completing the diary was the loss of their diaries by the participants. The health diary intervention significantly reduced reported healthcare expenditures. In the urban slum, average lab test expenditure dropped from 418 (36.9%) to four (0.3%), and travel expenses fell from 862 (76.1%) to six (0.5%). Similarly, in the rural area, lab test expenditure reduced from 235 (20.2%) to two (0.2%), and travel costs from 534 (45.9%) to zero, indicating improved cost-efficiency and care-seeking behavior. Utilization of government facilities for treatment in the urban (858, 75.7%) and rural sites (534, 45.9%) significantly reduced after the introduction of the health diary (Table 3).

**Table 3:** Change in reporting of illnesses and average monthly health expenses as a result of a study to determine health diary acceptability

Parameter		Urban		Rural	
		Baseline data n (%)	Diary data n (%)	Baseline data n (%)	Diary data n (%)
Illness reported		122 (10.7)	142 (12.5)	89 (7.6)	19 (1.6)
Monthly health expenses (Indian rupees)					
Medicine	(<5000 Rs)	0	8 (0.7)	0	3 (0.2)
Lab Test	(<5000 Rs)	418 (36.9)	4 (0.3)	235 (20.2)	2 (0.2)
Treatment cost	(≤5000 Rs)	3 (0.2)	0	0	0
	(>5000 Rs)	1 (0.08)	0	0	0
Utilization of government facility	(≤5000 Rs)	858 (75.7)	0	534 (45.9)	0
	(>5000 Rs)	5 (0.4)	0	0	0
Travel	(<5000 Rs)	862 (76.1)	6 (0.5)	534 (45.9)	0
Others expenses	(<5000 Rs)	0	60 (5.3)	0	0

Health parameter monitoring showed a notable shift in anemia, hypertension, and diabetes detection. Among urban men, the proportion of non-anemic individuals increased from 21 (1.8%) to 249 (21.9%), and in urban women, from 47 (4.1%) to 112 (9.8%). In rural men, non-anemic status rose from 18 (1.5%) to 327 (28.1%), and in rural women, from 45 (3.8%) to 402 (34.5%). Mild and moderate anemia detection improved concurrently. Reporting of re-hypertensive cases increased from 18 (1.5%) to 153 (13.5%) in the urban site and from 11 (0.9%) to 80 (6.8%) in the rural site. Similarly, hypertension reported in the urban site increased from 10 (0.8%) to 62 (5.4%) and from seven (0.6%) to 26 (2.2%) in the rural site, demonstrating the importance of the iceberg phenomenon of screening noncommunicable diseases in the community (Table 4).

**Table 4:** Distribution of health parameters in the study population

Parameters		Urban		Rural	
		Baseline n (%)	Health diary n (%)	Baseline n (%)	Health diary n (%)
Hemoglobin (five to 11 years)	Non-anaemic	23 (2.0)	42 (3.7)	16 (1.3)	122 (10.4)
	Mild	18 (1.5)	2 (0.1)	1(0.08)	0
	Moderate	5 (0.4)	8 (0.7)	5(0.4)	12 (1.0)
12 to 14 years	Non-anaemic	89 (7.8)	28 (2.4)	5 (0.4)	64 (5.5)
	Mild	2 (0.1)	2 (0.1)	0	10 (0.8)
	Moderate	7 (0.6)	10 (0.8)	0	7 (0.6)
Female (≥15 years)	Non-anemic	47 (4.1)	112 (9.8)	45 (3.8)	402 (34.5)
	Mild	33 (2.9)	40 (3.5)	16 (13.7)	115 (9.8)
	Moderate	28 (2.4)	138 (12.1)	25 (2.1)	192 (16.5)
	Severe	2 (0.1)	6 (0.5)	2 (0.1)	0
Men (≥ 15 years)	Non-Anaemic	21 (1.8)	249 (21.9)	18 (1.5)	327 (28.1)
	Mild	10 (0.8)	45 (3.9)	15 (1.2)	115 (9.8)
	Moderate	7 (0.6)	23 (2.0)	12 (1.0)	11 (0.9)
Hypertension	Normotensive	108 (9.5)	583 (51.5)	64(5.5)	655 (56.3)
	Pre-hypertensive	18 (1.5)	153 (13.5)	11 (0.9)	80 (6.8)
	Hypertensive	10 (0.8)	62 (5.4)	7 (0.6)	26 (2.2)
Diabetes	Non-diabetic	158 (13.9)	285 (25.1)	141 (12.1)	1152 (99.1)
	Prediabetic	2 (0.2)	26 (2.2)	1 (0.08)	2 (0.2)
	Diabetic	2 (0.2)	24 (2.1)	2 (0.2)	0

Cough, fever, and headache/fatigue were the most frequently reported symptoms in the urban and rural settings. Menstrual problems were reported to be more prevalent at the rural site than in the urban slum. Our health diary data show that functional intestinal disorders were most frequent in the rural area (Figure 2).

**Figure 3:**
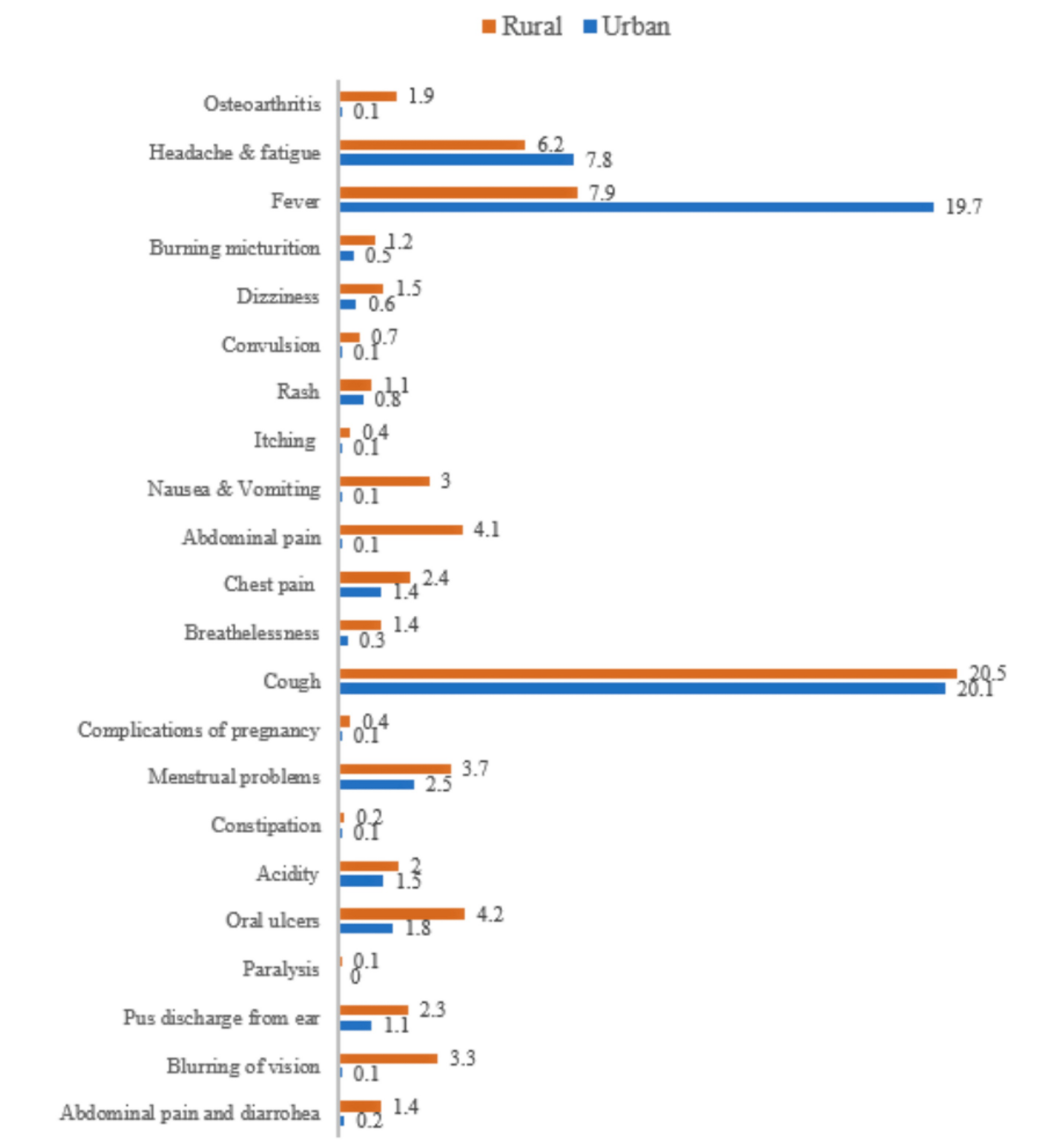
Percentage of illnesses reported in the two study sites

## Discussion

The findings of this study revealed that self-written health diaries are a feasible and acceptable tool for community-based health surveillance in both urban and rural settings in Central India. The high levels of participation and diary completion demonstrate a strong willingness among communities to engage in their own health monitoring, an observation supported by similar community-focused public health models elsewhere.

Acceptability and compliance

Despite challenges such as illiteracy (35% in both settings) and diary loss, the study achieved high diary retrieval and satisfaction rates. These outcomes align with the Community-Based Health Planning and Services (CPHS) program in Ghana, which emphasized that community ownership and ongoing engagement are critical to sustaining participation in community health initiatives [8]. Just as CPHS highlighted the importance of supportive supervision and continuous motivation, this study showed that study staff involvement (over 98% support in diary completion) was a key enabler of diary compliance.

Community surveillance and health awareness

A key finding in this study was the increase in health awareness and health-seeking behavior, as reflected by increased reporting of illnesses in the urban slum and improved documentation of conditions like anemia, hypertension, and diabetes. These observations are consistent with the Integrated Disease Surveillance and Response (IDSR) strategy implemented in Zambia, which emphasized that community participation and decentralized surveillance systems can help detect illnesses early and improve responsiveness to health issues. Mandyata et al. [9] noted that strong surveillance systems rely on timely data collection, community involvement, and efficient feedback loops. In our study, the consistent monthly collection of diary data and user feedback loops contributed to similar improvements in detection and awareness.

Health expenditure trends

The drastic reduction in reported health expenditures (e.g., lab test costs reduced from 36.9% to 0.3% in the urban slum) highlights how diary-based health monitoring may promote better use of public facilities and reduce unnecessary expenses. This mirrors the findings of Kweku et al. [8] in Ghana, where community-based planning reduced financial burdens and improved equity in health access. Moreover, this is in line with the broader goal of Universal Health Coverage (UHC), as discussed in several primary health care (PHC) initiatives in sub-Saharan Africa, where community-centered approaches resulted in lower out-of-pocket spending and improved access to preventive services [8].

Digital and logistical considerations

While our study used paper diaries, challenges with physical diary loss and long-term compliance highlight an opportunity to explore digital alternatives. The study by Kumar et al. [10] on the prevalence of digital communication tools in tribal, rural, and urban slum settings in India found an increasing penetration of mobile phones, even in low-income populations. This suggests that digital health diaries or mobile-based apps could serve as a scalable upgrade, especially for literate or semi-literate users.

Health outcomes and morbidity trends

Our study showed an increase in normotensive, pre-hypertensive, and hypertensive and non-diabetic, pre-diabetic, and diabetic classifications and improvements in hemoglobin levels, likely due to repeated health exposure and reminders via the diary. This echoes the findings from IDSR evaluations, where community-based documentation was seen to contribute to improved health outcomes by encouraging early diagnosis and continuous tracking [9]. Kweku et al. [8] revealed that Ghana's CPHS project identified health education, maternal care, and preventive services as areas that benefited significantly from consistent community interaction, a benefit our study also observed, particularly in awareness of menstrual disorders, oral hygiene, and anemia.

Limitations

One of the limitations of our study is recall bias. All diaries were updated monthly, and participants may not have remembered all health events or expenses accurately. Another limitation was the loss and damage of the diaries, leading to the non-retrieval of the recorded data. 

## Conclusions

The use of self-maintained health diaries in our study complements global evidence that community-based health monitoring tools are effective, empowering, and improve health literacy and service utilization. However, long-term success requires systemic support, digital evolution, and embeddedness in existing health systems. Future models may benefit from integrating digital diaries, ensuring strong community health worker engagement, and aligning such interventions with national surveillance frameworks like IDSR and National Health Mission (NHM).

To enhance the scalability and sustainability of health diary interventions, it is recommended that these tools be integrated with digital platforms such as mobile applications to enable real-time data synchronization and broader outreach. The training of community health workers, including Accredited Social Health Activists (ASHAs) and HHGs, is essential to provide ongoing support and supervision for diary use at the grassroots level. Incentivizing participation through health kits or formal recognition can further motivate consistent usage among community members. To institutionalize the intervention, it should be embedded within existing public health surveillance frameworks such as the Integrated Disease Surveillance Programme (IDSP) and Health and Wellness Centers. Finally, establishing regular feedback loops will ensure that the data generated from health diaries translates into actionable insights for localized planning and resource allocation, thus strengthening community-based health systems.
